# HBV/HIV Coinfection: Impact on the Development and Clinical Treatment of Liver Diseases

**DOI:** 10.3389/fmed.2021.713981

**Published:** 2021-10-04

**Authors:** Zhimeng Cheng, Panpan Lin, Nansheng Cheng

**Affiliations:** ^1^Department of Bile Duct Surgery, West China Hospital, Sichuan University, Chengdu, China; ^2^Laboratory of Aging Research and Cancer Drug Target, State Key Laboratory of Biotherapy and Cancer Center, National Clinical Research Center for Geriatrics, West China Hospital, Sichuan University, Chengdu, China

**Keywords:** hepatitis B virus, human immunodeficiency virus, coinfection, liver disease, clinical treatment

## Abstract

Hepatitis B virus (HBV) infection is a common contributor to chronic hepatitis, liver cirrhosis, and hepatocellular carcinoma. Approximately 10% of people with human immunodeficiency virus (HIV) also have chronic HBV co-infection, owing to shared transmission routes. HIV/HBV coinfection accelerates the progression of chronic HBV to cirrhosis, end-stage liver disease, or hepatocellular carcinoma compared to chronic HBV mono-infection. HBV/HIV coinfection alters the natural history of hepatitis B and renders the antiviral treatment more complex. In this report, we conducted a critical review on the epidemiology, natural history, and pathogenesis of liver diseases related to HBV/HIV coinfection. We summarized the novel therapeutic options for these coinfected patients.

## Introduction

Chronic hepatitis B virus (HBV) infection affects ~250 million people worldwide and can cause progressive liver fibrosis and hepatocellular carcinoma (HCC) (1, 2). Over 50% of HCC cases globally have been attributed to HBV infection (3, 4). Owing to their similar transmission patterns, HBV/HIV coinfection is relatively common in endemic areas (5). About 10% of HIV-infected individuals have been found to be chronically infected with HBV (6, 7). Compared with HBV mono-infection, HBV/HIV coinfection complicates the natural course and increases the risk of deterioration of diseases (8). Although some current therapeutic strategies are considered effective options in treating single virus infections, HBV/HIV coinfection has altered the natural history of the virus, requiring novel individualized therapeutic forms. This paper reviews the epidemiology, natural history, and pathogenesis of liver diseases in HBV/HIV coinfection. We also highlighted the individualized therapeutic options in these patients.

## Methodology

We performed a comprehensive literatures search in Pubmed, Embase, and Web of Science with the key words of “hepatitis B virus,” “HBV,” “human immunodeficiency virus,” “HIV,” “coinfection,” “liver disease,” “epidemiology,” “pathogenesis,” “treatment,” and “clinical treatment.” Clinical trials (http://clinicaltrials.gov/) was also searched for the important clinical trials about the HBV/HIV coinfection therapies. Literatures with no full text, describing only protocol design or preliminary results were excluded.

## Epidemiology

HBV, HIV, and HBV/HIV coinfection are caused by several means including unsafe drug injection, inappropriate medical practices, unsafe therapeutic injections, and high-risk unprotected heterosexual and man-man sexual acts. Overlapping transmission routes contribute to the prevalence of HBV, HIV, and HBV/HIV coinfection (9). Nevertheless, the prevalence varies in different geographic regions, ranging from 10 to 28% (10–13). Based on the prevalence of chronic HBV infection, it can classify the endemicity to high, intermediate, and low endemicity areas in geography (14). In the high endemicity area, such as sub-Saharan Africa and east Asia, ~10% of HIV-infected individuals have been observed to be concurrently infected with HBV (15). In Vietnam, the rate could be as high as 28% in the unsafe drug injection populations (11). In these regions, perinatal transmission, close household contact during childhood, or cultural procedures are the most common transmission routes (10, 16). However, in areas of low endemicity, such as North America, Western Europe, and Australia, HBV/HIV coinfection is usually recorded in adolescents or adults *via* unsafe drug injection or sexual transmission (15) with the estimated infection rate 6–14% (17). In the highest risk group of unsafe male homosexuals, the estimated rate ranges from 9 to 17% (14).

Nevertheless, prevalence of HBV/HIV infection showed a slightly decreased trend based on several studies published recently. An analysis developed by the North American Cohort Collaboration on Research and Design collected information covering 12 clinical sites from 1996 to 2010 and revealed that the prevalence of chronic HBV infection in HIV cohort was only 7% (18). Another research from the US Military HIV Natural History Study found that the overall incidence of chronic HBV infection was 4.3% (19). The most prevalence was appeared in 1995 with an obvious decrease in 2008, according to the evaluation of cross-sectional incidence (19). Even so, the health threat posed by HBV/HIV coinfection still cannot be ignored.

## Natural History

Acute HBV infection in adults is difficult to clinically detect. Spontaneously recovery is commonly observed among the most immunocompetent adults whose antibodies against hepatitis B surface antigen (anti-HBs) can be detected (20). Approximately 5–10% of immunocompetent adults will progress to chronic infection (21), 20% of individuals with chronic infection are likely to develop cirrhosis within 1–13 years (21). HCC and decompensated liver diseases occur in 6 and 23% of patients with cirrhosis, respectively (22).

The natural history of HBV factors are associated to the characteristics of virus, host, and environment. HBV/HIV coinfection accelerates the progression of HBV infection by impacting the immune response of the host (23). People infected with HIV have a high risk of contracting chronic HBV infection (24). Lower rates of HBeAg and/or HBcAg clearance and anti-HBe and anti-HBs seroconversion with higher rates of HBV replication were observed in HBV/HIV coinfected persons (25, 26). The acceleration of the process of cirrhosis and HCC is the most serious consequence in the liver-related damages (27). HBV/HIV-coinfected individuals have approximately five- to six-fold higher risk of HCC incidence with the presence of cirrhosis (28–30). Additionally, HIV/HBV-coinfected accelerate the progress to liver cirrhosis (31). Host CD4^+^ T cells are vital to the recognition of viral antigens presented by Kupffer cells and the regulation of the activities of CD8^+^ cytotoxicity T cells, antibody producing B cells, and secreting cytokines cells. Host immunosuppression as manifested by the depletion of CD4^+^ T cells may be the key for HIV to alter the natural course of HBV which associated to an increase in liver-related mortality (5, 32–34).

## Pathogenesis of Liver Diseases

The mechanism by which HIV facilitates liver-related damage has not been completely delineated. HIV-induced immunodeficiency seems to enhance HBV-related hepatotoxicity, which is mediated by the immune response (15). Depletion of CD4^+^T cells is an important feature of HIV infection which suppress the antigen presentation of liver resident macrophages (Kupffer cells) and the cytokine secretion of lymphocytes, in resulting the host immunosuppression (32). The inhibition of the host immune response enhances HBV replication substantially to further cause severe liver damage (35, 36). HBV infected hepatocytes is found non-cytopathic, without distinct cellular damage and viral cytopathic effects. However, HBV/HIV coinfection persons shows fibrosing cholestatic hepatitis (37, 38). HIV/HBV coinfection causes changes in the hepatic cytokine environment (15, 39, 40). It has been reported that HIV glycoproteins stimulate the hepatocyte to express the tumor necrosis factor related apoptosis inducing ligand (TRAIL) to induce hepatocyte apoptosis (41, 42). HIV envelope protein activates the caspase-independent apoptosis in Huh7 cells (43). HIV infection induces hepatocyte apoptosis through phagocytosed by macrophages or hepatic stellate cells and contributes to the inflammation and fibrosis of the liver (44). The increase of hepatocyte apoptosis has been observed among HBV/HIV coinfected patients compared with HBV mono-infected patients (45). HIV gp120 has been demonstrated to stimulate the hepatic expression of IL-8 to mediate the hepatic inflammation (46). Elevation of HBV load increases the X protein of HBV (HBx), which can also transactivate the expression of IL-8 *via* NF-κB and C/EBP-like cis-elements (47). As a leukocyte chemotactic molecule, IL-8 plays a crucial part in maintaining the inflammatory environment and HCC development (48). Furthermore, the content of IL-8 is positively correlated with the degree of liver damage (49).

HBx can also stimulate the expression of cyclooxygenase-2 (COX-2), which is overexpressed in liver cirrhosis (50, 51). Moreover, COX-2 expression can be activated by IL-8 through CREB and C/EBP (47). Accumulating evidence shows that HBV proteins activate IL-8 and COX-2 to maintain the inflammatory environment (47). The inflammatory hepatocytes secrete C-X-C motif chemokine 10 (CXCL10) which linked to the severity of liver damage involving viral hepatitis (52, 53). Once CXCL10 binds to its receptor, chemokine receptor 3 (CXCR3), immunocytes such as natural killer cells, and activated T cells and B cells are attracted to the inflammatory sites (54). Elevated CXCL10 was found in HBV/HIV coinfected patients but not in HBV mono-infected patients. This observation indicates that CXCL10/CXCR3 in the liver contributes to the acceleration of liver diseases (55–57).

In another hypothesis of mechanisms of the pathogenesis of liver diseases in coinfected patients, the depleted CD4^+^ T cells in the gastrointestinal tract contribute to the increase in microbial translocation and enhance the levels of circulating lipopolysaccharides (LPS) (58). When LPS binds to Toll-like receptor 4 and stimulates the NF-κB pathway or other pathways, it induces the secretion of pro-inflammatory cytokines (56). Although the relationship between microbial translocation and liver cirrhosis is reportedly closed, a similar evidence has never been found in HBV/HIV coinfected patients, including the direct relationship between circulating LPS and liver cirrhosis (56, 59). However, studies on simian immunodeficiency virus-infected rhesus macaques indicated that microbial load is capable of triggering the secretion of chemokines and enhancing the infiltration of CXCR6^+^-activated NK cells, thereby resulting in liver fibrosis (60). Further research is warranted to confirm this theory.

## Treatments Against HBV AND HIV

### Mechanism of Current and Experimental Dual Antiviral Therapies

Antiviral therapies should be initiated for HBV/HIV coinfected patients as soon as possible regardless of the clinical stage of the disease and the count of CD4^+^ cells (61, 62). This recommendation is based on the evidence that the effects of anti-HBV treatment might be reduced following the deterioration of immunodeficiency (63). Moreover, utilization of agents against HBV only can lead to drug resistance to HIV. Therefore, the optimal therapeutic options should include agents possessing dual anti-HBV and anti-HIV activity (64, 65). Current dual antiviral choices can be classified into virus-based agents and host-based agents. The former includes nucleoside/nucleotide reverse transcriptase inhibitors (NRTIs) and cyclophilin inhibitors. The latter consists of immunomodulators and monoclonal antibodies. Here, we provide an overview of the antiviral mechanism of these drugs.

**Figure 1:**
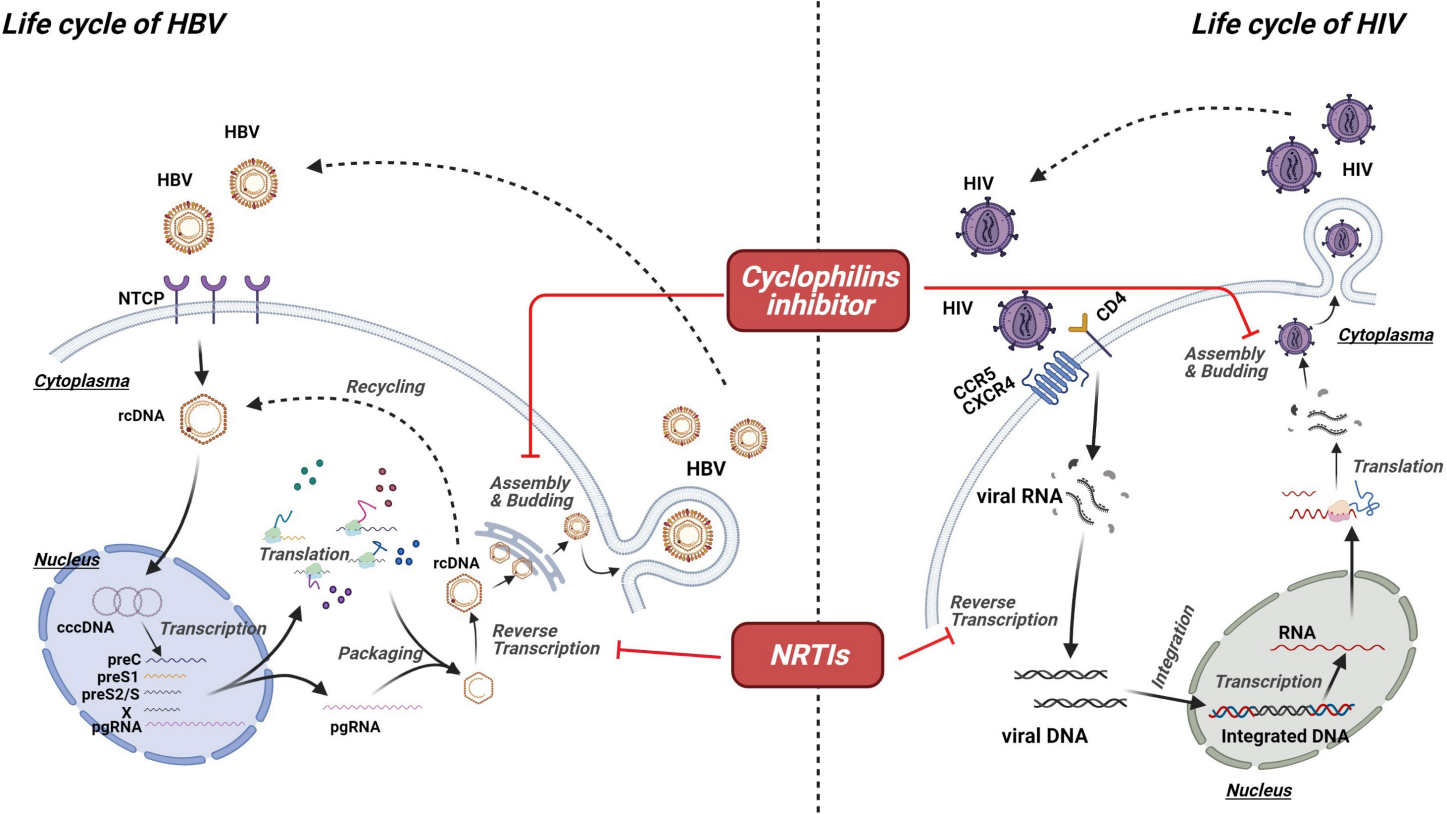
Mechanism of virus-targeted agents. Though undergoing different life cycles, HBV and HIV share some similarities in their life cycles, which are important for the development of dual antiviral drugs for HBV and HIV. Activated nucleoside/nucleotide reverse transcriptase inhibitors (NRTIs) are capable of disturbing the functions of both HIV reverse transcriptase and HBV polymerase by competing with natural nucleotide substrates for joining into DNA chains, resulting in the chain termination and viral replication. Cyclophilin A (Cyp A) plays a crucial role in the replication of various viruses, including HBV and HIV. Thus, cyclophilins inhibitor could be a potential option for anti-HIV and anti-HBV strategy.

The life cycle of a virus can be a potential target for antiviral agents. HIV is an RNA virus with the ability of reverse transcribing into DNA, which could be integrated into the host genome (66). By contrast, HBV is an enveloped DNA virus (67). Given that they belong to different types, HBV and HIV undergo different life cycles (Figure 1). Nevertheless, similarities in their life cycles are important in the development of dual antiviral drugs for HBV and HIV. According to a recent study, the polymerase of HBV and the reverse transcriptase of HIV have similar structures and functions, indicating that agents targeting these proteins have the ability to interrupt the life cycle of both HBV and HIV (68, 69). NRTIs are prodrugs that must be phosphorylated into active forms by cellular kinases (69, 70). Activated NRTIs are capable of disturbing the functions of both HIV reverse transcriptase and HBV polymerase by competing with natural nucleotide substrates for joining into DNA chains (69). In general, owing to the lack of 3′-OH, NRTIs work as chain-terminators, thereby interrupting DNA synthesis (69, 71, 72). Furthermore, protein priming activity, which is absent in HIV reverse transcriptase, is considered as another target for NRTIs (69). Inhibiting protein priming also substantially interferes with HBV replication (69). Cyclophilin A (Cyp A) belongs to the cyclophilin family with a peptidyl–prolyl isomerase activity (73, 74). Regarded as an acceleration factor for protein folding and assembly, Cyp A plays a crucial role in the replication of various viruses, including HBV and HIV. Moreover, it is linked to the pathogenesis of virus infection (75, 76). By interacting with the Gap protein of HIV or the small surface protein of HBV, Cyp A facilitates the replication and infectivity of viruses, suggesting that blocking Cyp A could be a potential anti-HIV and anti-HBV strategy (77, 78).

**Figure 2:**
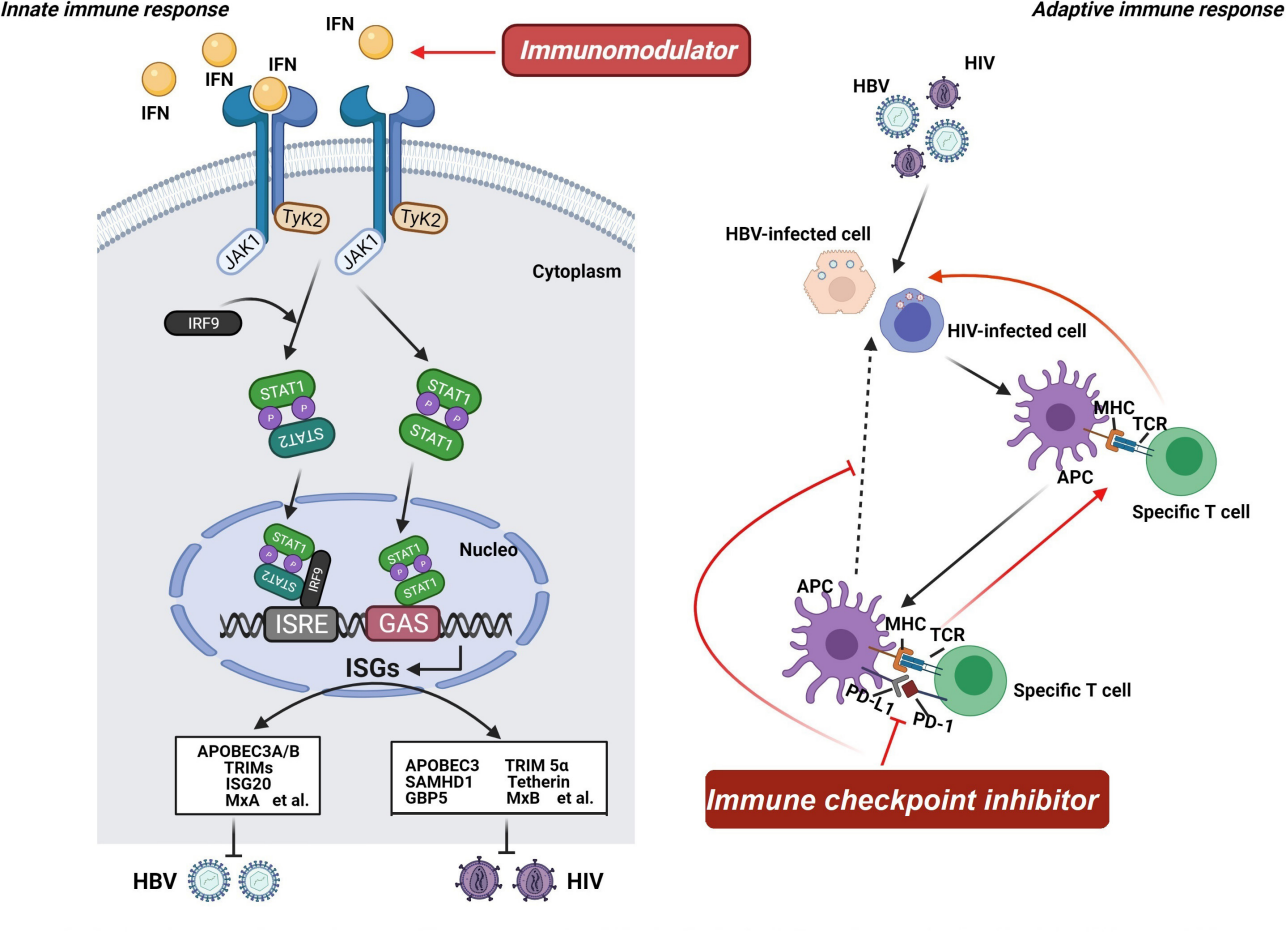
Mechanism of host-targeted agents. Pattern-recognition receptors are activated when the virus invades, leading to the production of interferon (IFN). IFN interacts with its cognate receptors and activates receptor-associated kinases, contributing to the activation of the STAT family to form a transcription factor complex or a homo-/heterodimer. The transcription factor complex and the homo-/heterodimer bind to the ISRE and GAS promoter elements, respectively, and encode numerous viral restriction factors with potent inhibition potential on viral replication. Consequently, immunomodulators offer a rational option for treating HBV and HIV infection. Additionally, the adaptive immune response is also a promising target for novel therapeutic interventions owing to the key position of T cells in viral infection control. Regardless of HBV or HIV infection, virus-specific T cells have been found to have a distinct dysfunction. And the PD-1/PD-L1 axis plays an important role in the pathogenic process of viruses. Considering that high expression levels of PD-1 and PD-L1 are usually related to unsatisfactory immune response, agents based on the PD-1/PD-L1 axis might be able to reverse this immune suppression consequence and exert corresponding antiviral effects. Hence, immune checkpoint inhibitor targeting the PD-1/PD-L1 axis can be another option for treating HBV/HIV coinfection.

In response to viral invasion, pattern-recognition receptors, including Toll-like receptors (TLRs), are activated, leading to the production of interferon (IFN) (79). Detection of viral DNA or RNA is crucial in triggering the innate immune response, culminating in the activation of transcription factors and release of antiviral cytokines, such as IFN (80, 81). When secreted, IFN interacts with its cognate receptors (i.e., IFNAR2) and activates receptor-associated kinases (i.e., JAK1 and Tyk 2), thereby contributing to the activation of the STAT family to form a transcription factor complex or a homo-/heterodimer (82–84). The transcription factor complex and the homo-/heterodimer bind to the ISRE and GAS promoter elements, respectively, and encode numerous viral restriction factors with potent inhibition potential on viral replication (Figure 2) (84–86). Consequently, immunomodulators (i.e., IFN), which can enhance the host's innate immune response, offer a rational option for treating viral infection, including HBV and HIV. The adaptive immune response is also a promising target for novel therapeutic interventions owing to the key position of T cells in viral infection control (66, 87). Regardless of HBV or HIV infection, virus-specific T cells have been found to have a distinct dysfunction, which is supposed to be linked to the expression disorder of programmed cell death protein (PD-1) and its ligand (PD-L1) (88). PD-1 and PD-L1 have been observed to be upregulated during viral infection, including HIV, HBV, and HCV, confirming that the PD-1/PD-L1 axis plays an important role in the pathogenic process of viruses (89). Considering that high expression levels of PD-1 and PD-L1 are usually related to unsatisfactory immune response, agents based on the PD-1/PD-L1 axis might be able to reverse this immune suppression consequence and exert corresponding antiviral effects, including on HBV and HIV (90, 91). Hence, immune checkpoint inhibitor targeting the PD-1/PD-L1 axis can be another option for treating HBV/HIV coinfection.

### Virus-Targeted Therapeutic Options

Specialized treatment and management of coinfected patients demand multidisciplinary cooperation. Although the life expectancy of HIV-infected individuals has been prolonged due to antiviral therapy (ART), liver injury induced by HBV has become the main cause of death in coinfected people. The primary objective of anti-HBV therapy is to suppress the replication of HBV, reduce the activity of inflammation, and halt the progression of liver damage. Several antiviral drugs have been approved for the clinical treatment of HBV, some of which are described as dual antiviral agents against HBV and HIV (Table 1). Combination antiviral therapies (cART) are commonly adopted in treating coinfected cases, and clinical trials have been conducted to explore their effectiveness in the treatment of coinfected populations (Table 2).

**Table 1:** Antiviral agents applicated in HBV/HIV coinfection.

**Type**	**Agent**	**Mechanism**	**Antiviral spectrum**	**Status**
Immunoregulator	Interferon	Inhibiting replication of HBV	HBV	Approved
	GS-9620	Antagonizing the TLR-7 and improving the host immune response, leading to the suppression of HBV and clearing of HIV.	HBV, HIV	Clinical trial
Nucleoside/Nucleotide reverse transcriptase inhibitor (NRTI)	Lamivudine	Terminating the chain and suppressing the replication of virus	HBV, HIV	Approved
	Emtricitabine	Terminating the chain and suppressing the replication of virus	HBV, HIV	Approved
	Tenofovir	Facilitating the HBeAg seroconversion of HBeAg and suppressing the replication of HBV	HBV, HIV	Approved
	Adefovir	Competing with deoxyadenosine triphosphate for integration in the synthesizing HBV DNA, resulting in the blockage of the viral DNA polymerase and termination of the chain	HBV	Approved
	Entecavir	Competing with guanosine for integration into the synthesizing HBV DNA, contributing to the blockage of viral DNA polymerase and chain termination	HBV	Approved
Cyclophilins inhibitor	CRV 431	Blocking the interaction of Cyp A with Gap protein of HIV as well as small surface protein of HBV, leading to the inhibition of viral replication.	HBV, HIV	Clinical trial
Immune checkpoint inhibitor	Pembrolizumab	Inhibiting the PD-1/PD-L1 axis and enhancing the immune response against virus infection.	HBV, HIV	Approved

*HBV, Hepatitis B virus; HIV, human immunodeficiency virus; TLR-7, Toll-like receptor-7; Cyp A, Cyclophilin A; PD-1, programmed cell death protein; PD-L1, programmed cell death protein ligand*.

**Table 2:** Important clinical trials of HIV/HBV therapies.

**Trial number**	**Phase**	**Status**	**Sample size**	**Design**
NCT01924455	IV	Completed	138	Maraviroc/Placebo
NCT00192595	IV	Completed	36	Tenofovir/Zidovudine, lamivudine, efavirenz
NCT01751555	IV	Completed	100	TDF/3TC/EFV
NCT03115736	II	Completed	24	Tenofovir Alafenamide
NCT03547908	III	Recruiting	240	B/FTC/TAF or DTG+FTC/TDF
NCT00476463	II	Completed	24	Emtricitabine
NCT03797014	IV	Recruiting	60	B/FTC/TAF
NCT00127959	IV	Completed	24	Tenofovir/emtricitabine/zidovudine/efavirenz
NCT03425994	–	Active, not recruiting	275	Elvitegravir/Cobicistat/Emtricitabine
NCT00033163	II	Completed	90	Adefovir dipivoxil/Tenofovir disoproxil fumarate
NCT00013702	II	Competed	30	Adefovir
NCT00023153	III	Completed	100	Adefovir dipivoxil
NCT01125696	II	Completed	45	Zidovudine/lamivudine/lopinavir-ritonavir or Tenofovir/lamivudine/lopinavir-ritonavir
NCT00391638	II/III	Completed	56	Peg-interferon Alpha 2a/Tenofovir /Emtricitabine
NCT02071082	III	Completed	79	E/C/FTC/TAF

*All the detailed information of clinical trials was registered on the website (ClinicalTrials.gov)*.

*TDF, tenofovir disoproxil fumarate; 3TC, lamivudine; EFV, efavirenz; B, bictegravir; FTC, emtricitabine; TAF, tenofovir alafenamide; DTG, dolutegravir; E, elvitegravir; C, cobicistat*.

#### NRTIs

##### Lamivudine

Lamivudine (3TC) is a dideoxynucleoside cytosine analog with an antiviral effect on both HBV and HIV (92). It exerts its antiviral ability by terminating the chain and suppressing the replication of virus, leading to the reduction in viral load and remission of disease symptoms (92, 93). Amelioration of liver fibrosis and suppression of liver disease progression can be achieved in patients with chronic HBV receiving lamivudine treatment (94–96). Furthermore, a remarkably improved virologic response was detected after 10 years of lamivudine treatment. HBV DNA was undetected among all patients, and 14 and 11% of patients achieved HBsAg seroconversion and loss, whereas 83 and 42% of patients achieved HBeAg seroconversion and loss, respectively (94).

As the first-line NRTI, lamivudine had been approved for HIV treatment in 1995 and had been proposed as part of fix-dose combinations in antiretroviral therapy (97, 98). Preclinical studies have revealed the potent antiviral efficacy of lamivudine; its half inhibitory concentration in infected cell lines of diverse HIV strains ranges from 0.002 to 1.14 μM (99, 100). Previous clinical trials also confirmed that single-tablet regimen (STR) containing lamivudine has persistent viral suppression and favorable safety in patients with HIV (93, 101). Dolutegravir is the most commonly used agent in combination with lamivudine for HIV clinical treatment. With regard to HBV/HIV coinfected individuals, lamivudine-based ART regimens achieved 30–60% HBV DNA suppression after 48 weeks of treatment (102–106).

Although lamivudine can be tolerated well and despite its outstanding antiviral effect, its application was restricted because of high rates of resistance, a character frequently observed in nucleoside analogs (107). Based on the guideline published in 2017 from the clinicalinfo.HIV.gov, lamivudine in combination with other antiviral agents, such as TAF or TDF, could be an alternative option for HIV-infected individuals with confirmed HBV infection (108). Nevertheless, regimens containing 3TC is hardly recommended to the treatment for coinfected patients, according to the latest guideline from British HIV association (BHIVA) and European AIDS Clinical Society (EACS) (109, 110).

##### Emtricitabine

Similar to lamivudine, emtricitabine (FTC) is a nucleoside with dual HBV/HIV inhibitory effects (111). Apart from HIV treatment, emtricitabine, as combined drug, has been approved by Food and Drug Administration (FDA) for the prevention of HIV infection (111). Although it is not an FDA-approved agent for HBV treatment, emtricitabine has an outstanding antiviral value against HBV; it can notably decrease HBV DNA in serum and achieve normal ALT in patients with HBV at the recommended dose of 200 mg/day (112). According to a preclinical study, emtricitabine is superior to lamivudine in terms of intracellular half-life (113). Nevertheless, both of them are considered clinically equivalent (114). A phase III clinical trial (NCT02607930) has demonstrated that emtricitabine-based STR was non-inferior in HIV virological suppression comparable to that of lamivudine-containing regimens (115). Moreover, this regimen involving emtricitabine, bictegravir, and tenofovir alafenamide affords guideline-recommended therapeutic strategy for HBV/HIV coinfected cohorts (115). Other clinical studies have also reached the same conclusion, confirming the clinical value of emtricitabine (114, 116).

Although limited studies are available to confirm the efficacy of emtricitabine in HIV/HBV coinfection cohorts, its combination with other antiviral agents are superior to emtricitabine monotherapy in reducing HBV DNA (117). A study also indicated that a combination of emtricitabine and tenofovir disoproxil fumarate (TDF) has excellent outcomes with 14% of patients achieved seroconversion to anti-HBe and 94% of them had undetected HBV DNA in the serum (118). Nevertheless, this study had a small sample size. Thus, multicenter and large-scale trials are needed to confirm the value of emtricitabine in HIV/HBV coinfection treatment. Currently, co-formulated FTC and TDF is recommended as part of a suppression combination regimen applicated in HIV-infected individuals with confirmed or presumed sensitive HBV (108, 109).

##### Tenofovir

Tenofovir is an adenosine nucleotide analog that has been approved for treatment of HIV infection (119, 120). Owing to its poor bioavailability, it is usually available commercially as TDF and tenofovir alfenamide (TAF) (119, 121). The former releases tenofovir in the bloodstream, whereas the latter releases tenofovir after entering the cells. Together with emtricitabine, tenofovir is used in HIV treatment and pre-exposure prophylaxis (PrEP) (122, 123). Furthermore, tenofovir-containing PrEP strategies are applicable for HIV-negative nursing mothers. In HBV treatment, tenofovir has been discovered to be capable of overcoming resistance to lamivudine and adefovir dipivoxil in HBV treatment (124, 125). Patients with HBV were found to benefit from tenofovir therapy, including TDF and TAF (126). Results showed that 73 and 75% of the HBeAg-positive individuals who received TDF or TAF achieved HBV DNA levels of <29 IU/mL at 96 weeks, respectively (126). Furthermore, no distinct difference was observed between TAF and TDF regimens in terms of loss rate and seroconversion of HBeAg and HBsAg (126). Two landmark studies also confirmed the therapeutic effects of TDF and TAF on HBeAg-positive or HBeAg-negative cohorts. Moreover, a low dose of TAF (25 mg/day) achieved a response similar to that of TDF (300 mg/day) at 48 weeks (127, 128). Akin to HBV, TAF exhibits potent anti-HIV effects *in vivo* at the low dose of 10 mg, which is 30-fold lower than that of TDF (129, 130).

Owing to their dual antiviral ability, tenofovir-containing regimens are extensively used to treat concurrent HBV and HIV (125, 131, 132). A study that enrolled 110 patients coinfected with HBV and HIV revealed that regimens containing TDF are superior to lamivudine therapy in the seroconversion rate of HBeAg (133). Result of this study showed that the proportion of patients displayed seroconversion was 57% in the group of TDF combined with FTC, 50% in the TDF group and 21% in the lamivudine group, respectively (133). Moreover, suppression of HBV replication was observed in 91% of the individuals during the median observation period. According to a meta-analysis of 23 studies involving a total of 550 patients with concurrent HBV and HIV and receiving TDF treatment noted persistent viral suppression, and the ratio of patients who achieved suppression of viral replication was 57.4, 79, and 85.6% after 1, 2, and 3 years, respectively (134). In addition, virus rebound had been rarely reported in TDF treatment. Therefore, all of the patients with HBV/HIV coinfection should receive tenofovir-based antiretroviral treatment unless history of tenofovir intolerance, according to the guideline of EACS (110).

Although rare, renal impairments, including tubular dysfunction, increase in serum creatinine, and acute renal failure, could be substantially induced by TDF. Hence, renal functions should be regularly monitored during TDF treatment (135).

##### Other NRTIs Used in HBV/HIV Coinfection Treatment

Not all NRTIs have potent antiviral effects on HBV and HIV. Apart from the agents discussed above, several other NRTIs, including adefovir, entecavir, and telbivudine, are active against HBV but display minimal activity against HIV (136–138). Consequently, their application in HIV treatment is rare. However, considering the potent suppressive effects of adefovir, entecavir, and telbivudine on HBV replication, experts proposed that these NRTIs might be of value in the treatment of HBV/HIV coinfection when combined with other antiviral agents.

As the first nucleotide analog approved for HBV treatment, adefovir strongly inhibits HBV replication with a low incidence of resistance (107). However, the dose of adefovir used in HIV treatment is usually linked to nephrotoxicity (31). According to a pilot study on HBV/HIV coinfection, adefovir can postpone the deterioration of liver diseases, enhance HBeAg seroconversion, and normalize ALT levels by suppressing HBV DNA (139). Several clinical trials have been conducted to estimate the value of adefovir in treating individuals with concurrent HBV and HIV (NCT00033163, NCT00013702, and NCT00023153). A prospective study (ACTG A5127) involving HBV/HIV coinfected patients revealed that either TDF or adefovir treatment results in evident decrease in serum HBV DNA; moreover, results showed that these NRTIs were well-tolerated (140). Benhamou (141) also indicated that treating with emtricitabine-containing regimen plus adefovir for 144 weeks decreased serum HBV DNA levels in 45% of HBV/HIV coinfected subjects, which was lower than that in HBV monoinfection (56%).

Entecavir, a guanosine analog, is superior to emtricitabine and adefovir in suppressing serum HBV DNA (142–144). Moreover, it is effective against not only wild-type HBV but also emtricitabine-resistant and adefovir-resistant HBV (31). Although entecavir was once considered as an inactive agent to HIV, a study uncovered a remarkable phenomenon showing that entecavir could result in evident reduction in serum HIV RNA in three HBV/HIV coinfection patients (145). However, such residual antiviral activity might be able to induce resistant changes in HIV (71). Hence, the FDA warned that entecavir should not be used in the absence of antiretroviral therapy in HBV/HIV coinfected cohorts (137). Numerous clinical trials have been conducted to explore the potential value of entecavir in treating patients coinfected with HBV and HIV (Table 2).

#### Cyclophilin Inhibitors

##### CRV 431

CRV431, which was previously called CPI-431-32, is a non-immunosuppressive cyclophilin inhibitor–cyclosporin A analog (146). Previous studies have confirmed the efficacy of cyclophilin inhibitors against HIV and HCV (146, 147). HBV DNA, HBsAg, and HBeAg could be effectively reduced by CRV 431 by interrupting the interaction of CypA with HBsAg or HBeAg, as well as by blocking the entry of HBV (148). In addition, an *in vivo* study of transgenic mice reported that CRV 431 can lower serum HBsAg levels and HBV DNA loads in the liver in a dose-dependent manner (149). Moreover, the viral inhibitory effect was enhanced when low CRV 431 dose (10 mg/kg/day) was added 5 mg/kg/day of tenofovir exalidex, a prodrug of tenofovir (149). Furthermore, liver fibrosis and tumor burden in a non-alcoholic steatohepatitis mouse model were ameliorated, highlighting the potential of CRV 431 as a novel therapy for liver disease (150). An ongoing clinical trial is assessing the safety and tolerability of CRV 431 in healthy volunteers (NCT 03596697).

CsA analogs have been confirmed to be effective against HIV by blocking cyclophilins and the HIV capsid to form complexes (151, 152). Hence, CRV 431, which belongs to CsA analogs, could be another promising agent with anti-HIV activity. According to its metabolization, CRV 431 is speculated to not interact with other NRTIs (153). Therefore, CRV431 might be a potential agent for the treatment of patients suffering from both liver diseases and HIV. Coformulation of CRV 431 and current drugs could achieve favorable outcomes in HBV/HIV coinfected patients.

### Host-Targeted Therapeutic Options

#### Immunoregulator

##### Interferon-α and PEGylated Interferon-α

Interferons (INFs), a cluster of signaling proteins, are secreted by host cells in response to pathogenic invasion (154, 155). Moreover, they are the first class of agents approved for the treatment of chronic hepatitis B. Interferon-α (INF-α) used to be the standard choice but was eventually replaced by PEGylated interferon-α because the latter has a longer half-life and a stronger potency than the former (31, 156). INF-α and PEGylated INF-α can effectively inhibit HBV replication *in vitro via* stimulated-INF genes and augment host immunity to defend against HBV infection. IFN therapy has shown remarkable efficacy among HBeAg+ HBV infection patients with the characteristic of elevated alanine aminotransferase and low serum HBV DNA (157–159). Nevertheless, limited benefits and increased toxicity were discovered in HIV/HBV coinfected patients probably because of abnormal immunity (25, 160). Hence, such agents might be applied to non-decompensated patients who have a good response to INFs (161). In general, the period of treatment lasts for 12 months. The guideline of BH *via* in 2013 gave the recommendation that PEG-IFN should be used only in HBsAg+ individuals with repeatedly raised ALT and low level of HBV DNA, regardless of the status of HBeAg (162). Nevertheless, the place of PEG-IFN therapies is not mentioned in neither the latest EACS nor BHIVA.

##### GS-9620

GS-9620, also known as vesatolimod, is an oral small-molecule antagonist of toll-like receptor 7 (TLR-7) with outstanding anti-HBV potency; moreover, it is considered in the clinical treatment of chronic HBV infection (163, 164). Preclinical studies reported sustained reduction of HBV DNA, HBV RNA, and HBsAg levels in HBV-infected cells administered with GS-9620 through an IFN-dependent manner (163, 165, 166). According to a study on a chimpanzee model of chronic HBV, GS-9620 participates in the accumulation of CD8^+^ T cells and B cells in the portal regions of liver, thereby playing a role in wiping out HBV-infected cells or restricting HBV infection (167). Another study on woodchucks with chronic HBV infection found reduced levels of cccDNA and risk of HCC due to GS-9620 (165). Having achieved favorable HBV suppressive effects in preclinical studies, GS-9620 is undergoing clinical trials to definitively establish its therapeutic efficacy in patients with chronic HBV. Currently, GS-9620 is considered to be safe in and well-tolerated by individuals with chronic HBV (168–170).

Existing HIV therapeutic strategies can achieve HBV suppression to undetectable levels rather than complete removal of viruses, which leads to lifelong treatment (171). To overcome this difficulty, experts have focused on the induction of latent HIV expression and the enhancement of the viral recognition ability of the immune system to eliminate latent HIV. On the basis of the viewpoint that TLR can induce HIV expression from infected cells, scientists have explored the value of GS-9620 in HIV treatment (171–173). Results were consistent with this assumption, confirming that GS-9620 has the ability to activate HIV from the peripheral blood mononuclear cells of HIV-infected patients receiving anti-HIV treatment, thereby improving immune functions and enhancing HIV clearance (171). HIV replication was also observed during GS-9620 treatment (174). A clinical trial (NCT02858401) reported that GS-9620 is safe in and well-tolerated by HIV-infected cohorts (175). Owing to its potential dual antiviral capability, GS-9620 is a promising novel agent that can be applied in the combination therapy of HBV/HIV coinfection.

#### Immune Checkpoint Inhibitor

##### Pembrolizumab

Pembrolizumab, a checkpoint inhibitor that targets PD-1, has been approved for treatment of various carcinomas, including lung cancer (176). On account of the critical function of the PD-1/PD-L1 axis in the pathogenesis of chronic diseases, including HBV and HIV, PD-1 inhibitors have been speculated to be effective in disease treatment (90, 91, 177, 178). Several studies have evaluated the safety and feasibility of pembrolizumab in the therapy of patients suffering from various types of carcinoma concurrently with HIV (179–182). Treatment with pembrolizumab has an impact on HIV-specific T cell response and HIV load, showing as a transient increase of CD8^+^ T cell activation and a transient reduction of HIV DNA (180, 182). No serious adverse effects were observed during the treatment, indicating that pembrolizumab is safe and well-tolerated by the patients (181). Among patients with tumor and HBV infection, pembrolizumab was found to be safe (183–185). Moreover, several studies have been developed to explore the efficacy of pembrolizumab in HBV infection, suggesting that it might enhance the host immune status (182, 184, 185). However, further research is needed to assess the efficacy and safety of pembrolizumab in HBV treatment. On the basis of the application of pembrolizumab in the treatment of patients with cancer and HIV or HBV, experts assumed that it might be effective in HVB/HIV coinfected individuals. However, the evidence remains inadequate because patients with HIV or HBV are usually excluded from research on immune inhibitors because of the immune reconstitution inflammatory syndrome (186). Few studies have indicated that PD-1 inhibitors have proviral effects on HBV infection. However, they are regarded to be able to strengthen the immune function and may be a potential option for HIV treatment (88). Combination of PD-1 inhibitors with other agents might be a reasonable strategy for viral coinfection treatment.

### Immune Reconstitution Inflammatory Syndrome

Though current guidelines suggest treatment of HBV/HIV coinfected patients with dual antiviral regimen targeting HBV and HIV, immune reconstruction-related hepatic flare following the ART should be noted (187). IRIS is considered as a complication induced by the initiation of highly active antiretroviral therapy (ART) in HBV/HIV coinfected patients. It is an inflammatory disorder related to the worsening status of existing infection (188).

As to the coinfected patients receiving ART, elevation of liver enzymes is common, most of which are mild and do not require modification of treatment (189, 190). It is uncommon to develop into severe hepatotoxicity, manifesting as liver enzymes higher than 10 times the upper limit of normal (191). Moreover, the acute liver failure is also rare (192). Unfortunately, high proportion of mortality can be observed among acute liver failure patients, maybe owing to the potential impairment of liver (191, 193). According to a recent research, 20–25% of the coinfected patients might appear HBV flares (HF) after the start of ART (194). Currently, little is known about the impact of IRIS induced HF on the natural history of HBV infection. Patients undergoing HF presented an increase of CD4 T cell counts, a peak level of serum alanine aminotransferase (ALT) and a decrease of HBV DNA (195, 196). A recent study revealed that HBsAg loss was more common in patients developed IRIS induced HF compared with those who did not, suggesting that IRIS induced HF after ART was closely linked with the loss of HBsAg (197). The researchers also raised that younger age and higher HBV DNA titer at baseline were related with the development of IRIS induced HF (197). However, the occurrence mechanism of HF has never been illustrated clearly. It is speculated that the exploration of immune response of IRIS induced HF might be benefit to the treatment of HBV/HIV coinfection. Further studies on such aspect are warranted.

## Conclusion

Accumulating evidence indicates that the coinfection of HBV and HIV place a heavy burden to the society (148, 198, 199). Coinfection is capable of accelerating the progression of liver diseases (200). Treatment with dual antiviral agents must be initiated as soon as possible (61, 62). However, several factors increase the difficulty of treatment. Agents with a single antiviral effect could induce drug resistance during the duration of therapy. For instance, agents against HBV only could lead to drug resistance to HIV. Hence, combination therapeutic strategies with dual antiviral effects are important (65). Drug-related side effects must also be considered when formulating therapeutic regimens. Renal dysfunction is the most common adverse effect, thus it should be considered before choosing drugs, especially tenofovir, for treatment (135). Damages to important organs might limit the application of existing regimens. Therefore, novel dual antiviral agents with less adverse effects must be developed. In-depth research on disease mechanisms has identified several critical pathogenic mechanisms, providing new approaches for disease treatment. PD-1/PD-L1 participates in the pathogenicity of viruses, including HBV and HIV (88–90). The inhibitors that antagonize the PD-1/PD-L1 axis might be a promising drug for HBV/HIV coinfection treatment. Moreover, immunoregulators with the ability to enhance the innate immune response against HBV and HIV are acceptable. Regardless of the type of agents applied for the treatment of HBV/HIV infection, drug-related adverse effects should be closely monitored.

The efficacy and safety of many strategies for the treatment of HBV/HIV coinfection are being assessed in clinical trials. Several agents remain at the preclinical phase and are not yet available for the clinical treatment of HBV/HIV coinfection. More research and clinical trials are required to definitively establish the value of such agents for the therapy of HBV/HIV coinfection. Finally, novel agents with potent antiviral effects on both HBV and HIV are the ideal approaches.

## Author Contributions

All authors listed have made a substantial, direct and intellectual contribution to the work, and approved it for publication.

## Conflict of Interest

The authors declare that the research was conducted in the absence of any commercial or financial relationships that could be construed as a potential conflict of interest.

## Publisher's Note

All claims expressed in this article are solely those of the authors and do not necessarily represent those of their affiliated organizations, or those of the publisher, the editors and the reviewers. Any product that may be evaluated in this article, or claim that may be made by its manufacturer, is not guaranteed or endorsed by the publisher.
